# MoS_2_ Nanosheets Assembled on Three-Way Nitrogen-Doped Carbon Tubes for Photocatalytic Water Splitting

**DOI:** 10.3389/fchem.2019.00325

**Published:** 2019-05-17

**Authors:** Yujia Zhang, Yan Liu, Wen Gao, Ping Chen, Hongyu Cui, Yanfei Fan, Xifeng Shi, Yingqiang Zhao, Guanwei Cui, Bo Tang

**Affiliations:** Key Laboratory of Molecular and Nano Probes, Ministry of Education, Shandong Provincial Key Laboratory of Clean Production of Fine Chemicals, College of Chemistry, Chemical Engineering and Materials Science, Collaborative Innovation Centre of Functionalized Probes for Chemical Imaging in Universities of Shandong, Shandong Normal University, Jinan, China

**Keywords:** photocatalysis, MoS_2_, three-way nitrogen-doped carbon tubes, hydrogen evolution, water splitting

## Abstract

In this work, a micron-sized three-way nitrogen-doped carbon tube covered with MoS_2_ nanosheets (TNCT@MoS_2_) was synthesized and applied in photocatalytic water splitting without any sacrificial agents for the first time. The micron-sized three-way nitrogen-doped carbon tube (TNCT) was facilely synthesized by the calcination of commercial sponge. The MoS_2_ nanosheets were assembled on the carbon tubes by a hydrothermal method. Compared with MoS_2_, the TNCT@MoS_2_ heterostructures showed higher H_2_ evolution rate, which was ascribed to the improved charge separation efficiency and the increased active sites afforded by the TNCT.

## Introduction

Photocatalytic water splitting is one of the promising strategies to address the global energy and environmental problems (Hinnemann et al., 2005; Dong et al., 2013; Jiang et al., 2013; Chang et al., 2014; Pan et al., 2016; Wang et al., 2016; Anna et al., 2018; Chen et al., 2018; Zeng et al., 2018). TiO_2_ is the most investigated material in the semiconductor photocatalysis research field. However, due to the wide bandgap, it is only responsive to UV light, which greatly limits its photocatalytic efficiency (Cui et al., 2013; Shi et al., 2018). In recent years, many visible-light-driven semiconductors have been designed and applied in solar energy conversion research fields (Wang et al., 2014; Pan, 2016; Shao et al., 2017, 2018; Cheng et al., 2018, 2019; Marchal et al., 2018; Wolff et al., 2018; Yang et al., 2019). Molybdenum disulfide (MoS_2_) is one of the most attractive materials. As a graphene-like hexagonal material with close-packed layered structure, MoS_2_ has a sandwich architecture, in which the strong covalent bonds among S-Mo-S atoms lies in the layer while the weak van der Waals force exists between the layers (Cheiwchanchamnangij and Lambrecht, 2012). With the variable atomic coordination and the electronic structure, MoS_2_ exhibits extremely fast carriers mobility (over 200 cm^2^·V^−1^·s^−1^). Furthermore, the band gap is adjustable from 1.19 to 1.80 eV through the variation of layer thickness, nanometer size and ion doping. Hence, MoS_2_ is an excellent light absorbing material and has high utilization of sunlight. Additionally, the nano-scale molybdenum disulfide has a complicated edge structure with high unsaturation and high reactivity (Wang et al., 2018). In a word, MoS_2_ has optimal band gap, high reactive spots and fast mobility of charge carriers, which is beneficial for the photocatalysis. However, the photocatalytic efficiency of pure MoS_2_ is still limited by the fast recombination of photogenerated carriers. The construction of heterostructure of nano-sized MoS_2_ coupled with other semiconductor or carbon materials has attracted great interest (Xiang et al., 2012; Jia et al., 2013; Guo et al., 2015; Lang et al., 2015; Pan et al., 2016). It was proposed that the hybrids could provide appropriate band structure for water splitting and improve the separation efficiency of photogenerated carriers.

**Scheme 1:**
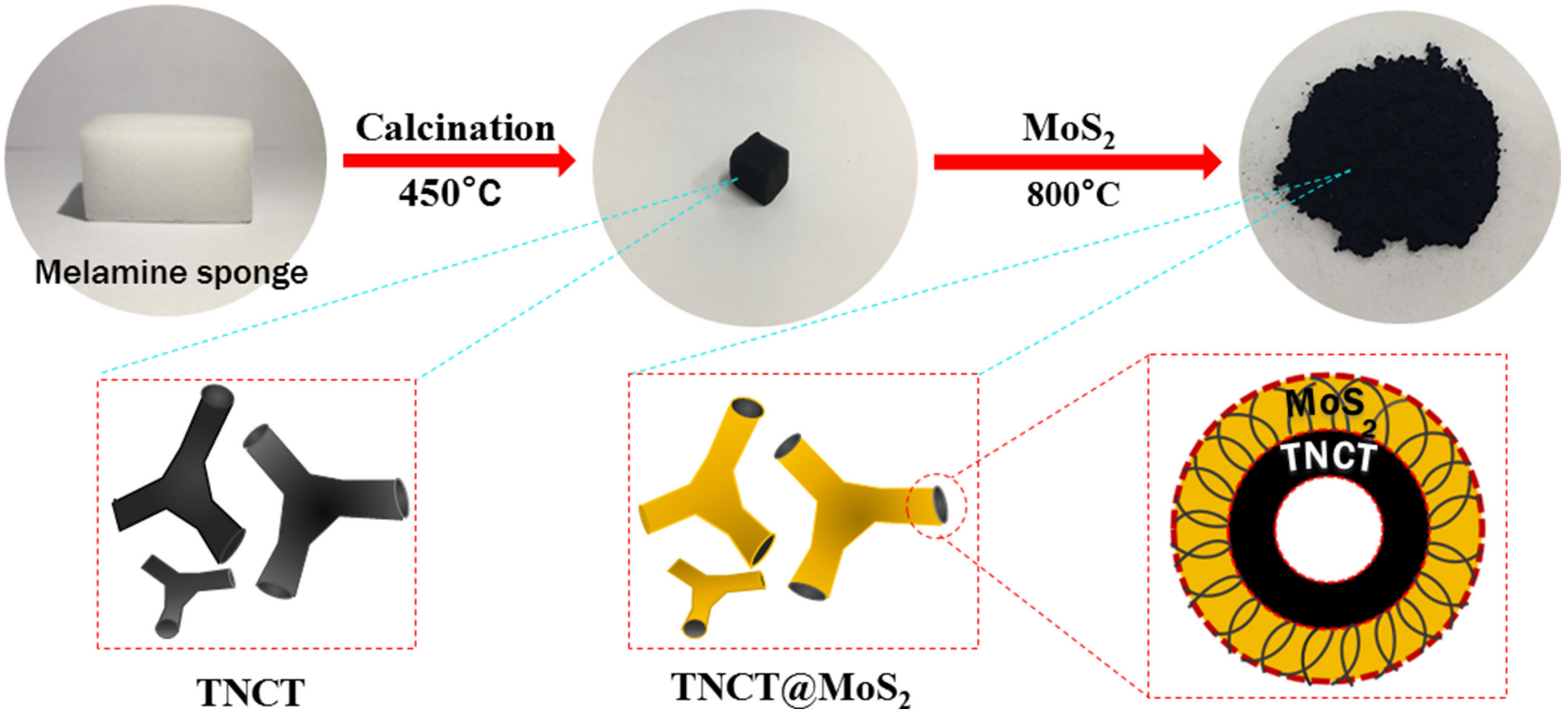
Illustration of the synthesis process of TNCT@MoS_2_.

Herein, MoS_2_ nanosheets assembled on a micron-sized three-way nitrogen-doped carbon tube (TNCT@MoS_2_) was synthesized and applied in the photocatalytic water splitting for the first time. As illustrated in Scheme 1, the micron-sized three-way nitrogen-doped carbon tube (TNCT) was first prepared by a calcination method. Then, the MoS_2_ nanosheets were loaded on the TNCT by a hydrothermal method. The as-prepared TNCT@MoS_2_ composite exhibits much higher photocatalytic activities than pure MoS_2_, which is ascribed to the improved charge separation and transfer efficiency afforded by the TNCT.

## Experimental Section

### Materials

Ammonium molybdate tetrahydrate, thiourea, ethanol, and terephthalic acid (TA) were supplied by China National Pharmaceutical Group Chemical Testing Co., Ltd. Melamine sponge is purchased from Zhengzhou Fengtai nanomaterial Co., Ltd. The water used in the experiment is secondary deionized water.

### Preparation of TNCT

The commercial melamine sponge was calcined at 450°C for 3 h in vacuum, and the sponge changed from white to black. At this time, the required three-way nitrogen-doped carbon tube (TNCT) was initially formed.

### Fabrication of TNCT@MoS_2_

First, 1.24 g ammonium molybdate tetrahydrate, 2.26 g thiourea and 35 ml deionized water was mixed and stirred at room temperature for 20 min. Then, the mixture and the as-prepared black TNCT were transferred to a 100 mL polytetrafluoroethylene reactor. The reactor was heated in an oven at 220°C for 24 h. After the reaction, the supernatant is poured off, and the sponge block is clamped out. After being mashed, the sponge block was centrifuged and washed with distilled water and ethanol for several times. Then, it was dried in a vacuum drying box at 60°C for 12 h, and a black powder was obtained. Finally, the black powder was calcined at 800°C in N_2_ atmosphere for 4 h.

### Instruments

Scanning Electron Microscope (SEM) equipped with a field-emission gun operated at 5.0 kV was used to characterize the morphology of the as-obtained product. High-Resolution Transmission Electron Microscopy (HRTEM) was taken on JEM-2100F instrument at an accelerating voltage of 200 kV. X–ray diffraction (XRD) analyses carried out on a Bruker D8 Advance Diffractmeter with Cu Kα radiation (1.5418 Å). X-ray photoelectron spectroscopy (XPS) was carried on Thermo Scientific Escalab 250Xi with Al Kα as the excitation source. Photoelectrochemical performance measurements were performed in a standard three-electrode PEC cell, with three-way nitrogen-doped carbon tube@MoS_2_, saturated calomel electrode, and Pt wire as the working electrode, reference electrode, and counter electrode, respectively. The fluorescence spectrum was carried out with an Edinburgh FLS920 spectrofluorimeter (Edinburgh Instruments Ltd, England) equipped with a xenon lamp. Raman spectra were measured by LabRAM HR800 confocal microscope Raman spectrometer from Horiba Jobin-Yvon, France. The UV/Vis diffuse reflection spectra (DRS) were taken on a Shimadzu UV-2550 spectrophotometer with an integrated sphere attachment and BaSO_4_ used as the reference sample.

### Photocatalytic Activity for Water Splitting

In a typical process, 20 mg of the as-prepared photocatalysts and 10 mL aqueous solution in a 20 mL Quartz bottle sealed with silicone rubber septum. Prior to photocatalysis experiment, the sample solutions were thoroughly deaerated by evacuation and purged with nitrogen for 10 min. Then it was irradiated by a 1,000 W Xenon lamp (the light intensity irradiating the photocatalysts was 0.17 W) at room temperature under constant stirring. The produced gas was analyzed by gas chromatography (FULI 9750, TCD, Nitrogen as the carrier gas, and 5 Å molecular sieve column).

Because there was no O_2_ detected in this photocatalytic water splitting system, to confirm the photocatalytic water splitting process, the intermediate OH directly photogenerated from water was determined as following (Yang et al., 2009). A fluorescence probe named terephthalic acid (TA) was added into the above-mentioned photocatalytic H_2_ evolution measurement system to capture OH photogenerated from water, in a result of producing TAOH. Typically, 0.01M TA, 20 mg TNCT@MoS_2_, and 10 mL water were mixed in a 20 mL Pyrex bottle at ambient temperature and atmospheric pressure, sealed with silicone rubber septum. The sample solutions were thoroughly deaerated by evacuation and argon bubbling for 2 h prior to photocatalysis experiment. Then it was irradiated by a 1,000-W Xe lamp under ambient conditions and magnetic stirring for certain time. The produced solutions containing TAOH were analyzed by fluorescence spectra.

## Results and Discussion

**Figure 1:**
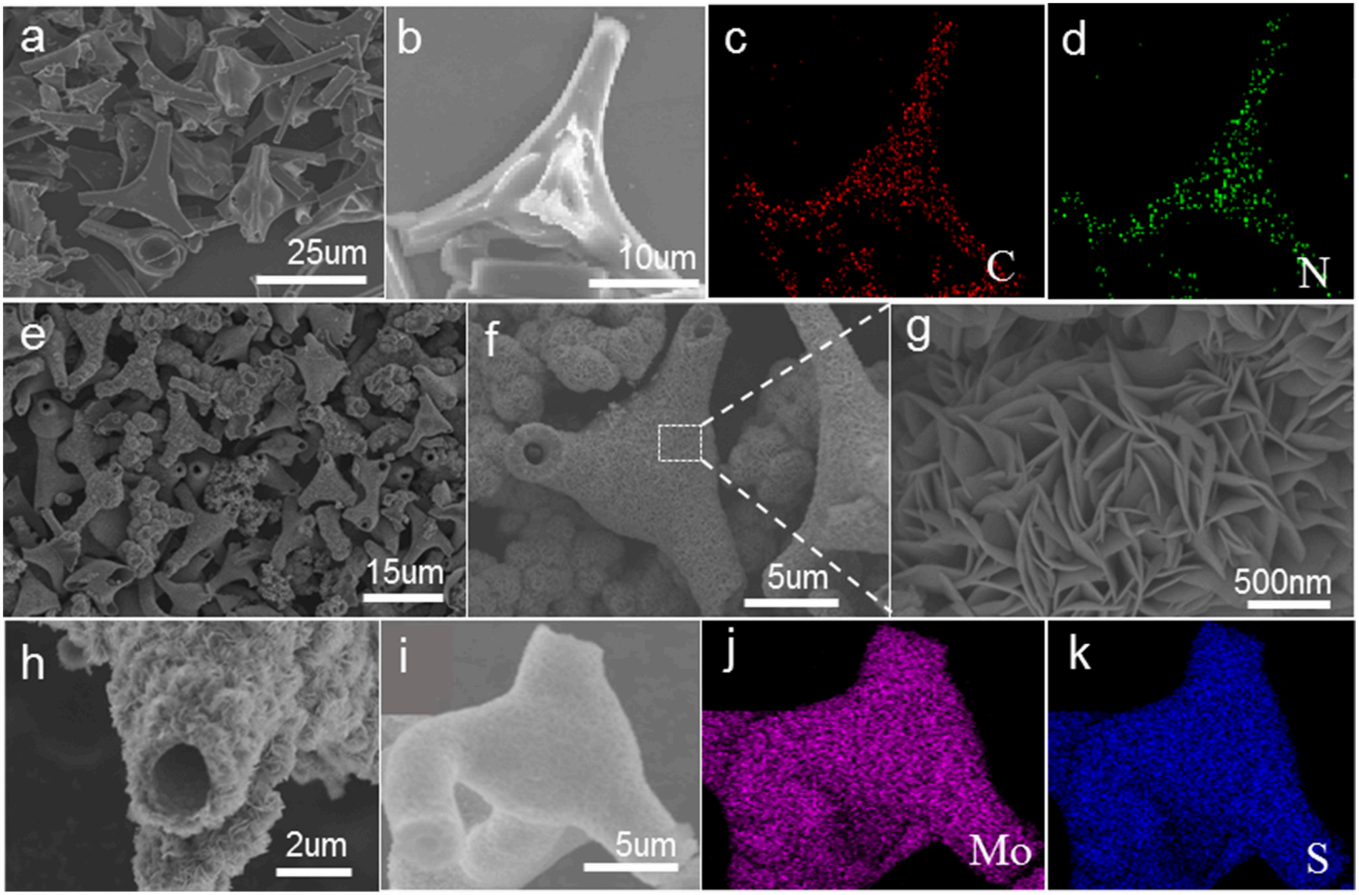
SEM images of TNCT **(a,b)** and TNCT@MoS_2_ **(e–i)**. The down two panels shows the element mapping images of C,N of TNCT **(c,d)** and Mo, S of TNCT@MoS_2_ **(j,k)**.

As shown in Figures 1a,b, most of these nitrogen-doped carbon tubes have three-way branches, each of which is about 20 microns in length and 3 micron in diameter. The EDS-mapping pictures show that the doped nitrogen elements are uniformly dispersed in the carbon tubes (Figures 1c,d). The SEM image of TNCT@MoS_2_ heterostructures is shown in Figures 1e–h. It can be seen that the MoS_2_ nanosheets layer with thickness of 500 nm are uniformly coated on the TNCTs. The MoS_2_ nanosheets are interconnected with each other, forming the 2D nanosheets networks. Additionally, the EDS mapping pictures show that MoS_2_ nanosheets are uniformly dispersed on the surface of TNCT (Figures 1j,k).

**Figure 2:**
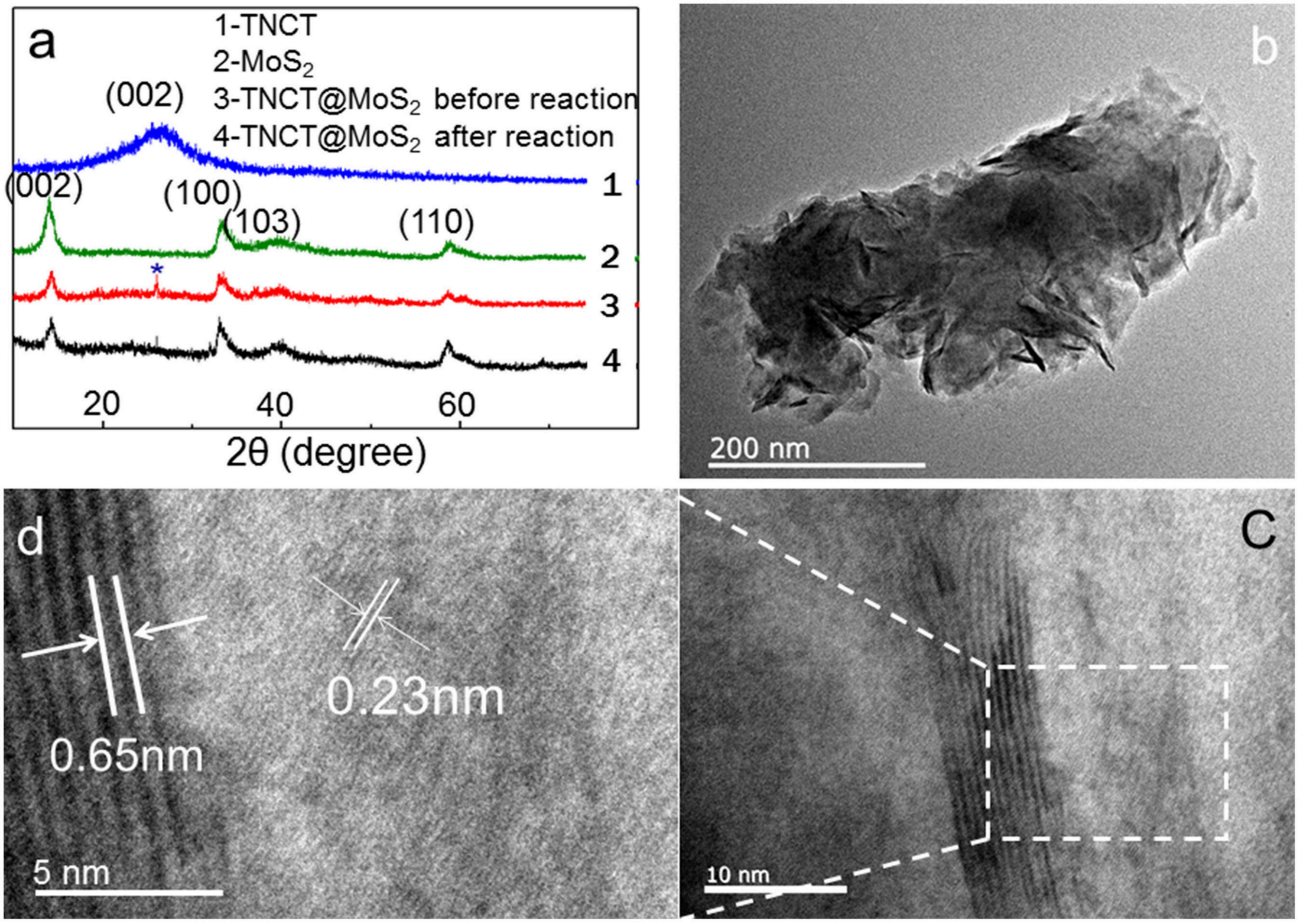
XRD **(a)**, HRTEM **(b–d)** patterns of TNCT@MoS_2_. * indicates the (002) crystal plane of TNCT.

XRD peaks of TNCT@MoS_2_ localized at 2θ values of 13.8, 33.5, 39.8, and 58.8° are ascribed to MoS_2_ with typical hexagonal phase(JCPDS:00-037-1492, Figure 2a). The (002) crystal plane diffraction peak is stronger than the diffraction peaks on other crystal planes, indicating that MoS_2_ has a good layered packing structure (Zong et al., 2008). An obvious reflection located at 2θ = 26.5°is ascribed to the TNCT (002) crystal plane (Figure 2a; Tang et al., 2015). The heterogeneous lattice interfaces of MoS_2_ and TNCT were clearly observed on the HRTEM images (Figures 2b,d). The lattice fringe spacing of 0.65 nm is ascribed to hexagonal phase MoS_2_ (Choi et al., 2017). The lattice fringes of TNCT are not significant maybe due to the doping of nitrogen atoms, a dimly visible lattice spacing of 0.22 nm was ascribed to the (100) facet of graphite (Baker and Baker, 2010). The strong wide peaks located at 1,379 cm^−1^ (D-band) in the Raman spectra showed that the presence and partial graphitization of carbon (Supplementary Figure 1; Matthews et al., 1999) in agreement with the results observed from the HRTEM images.

**Figure 3:**
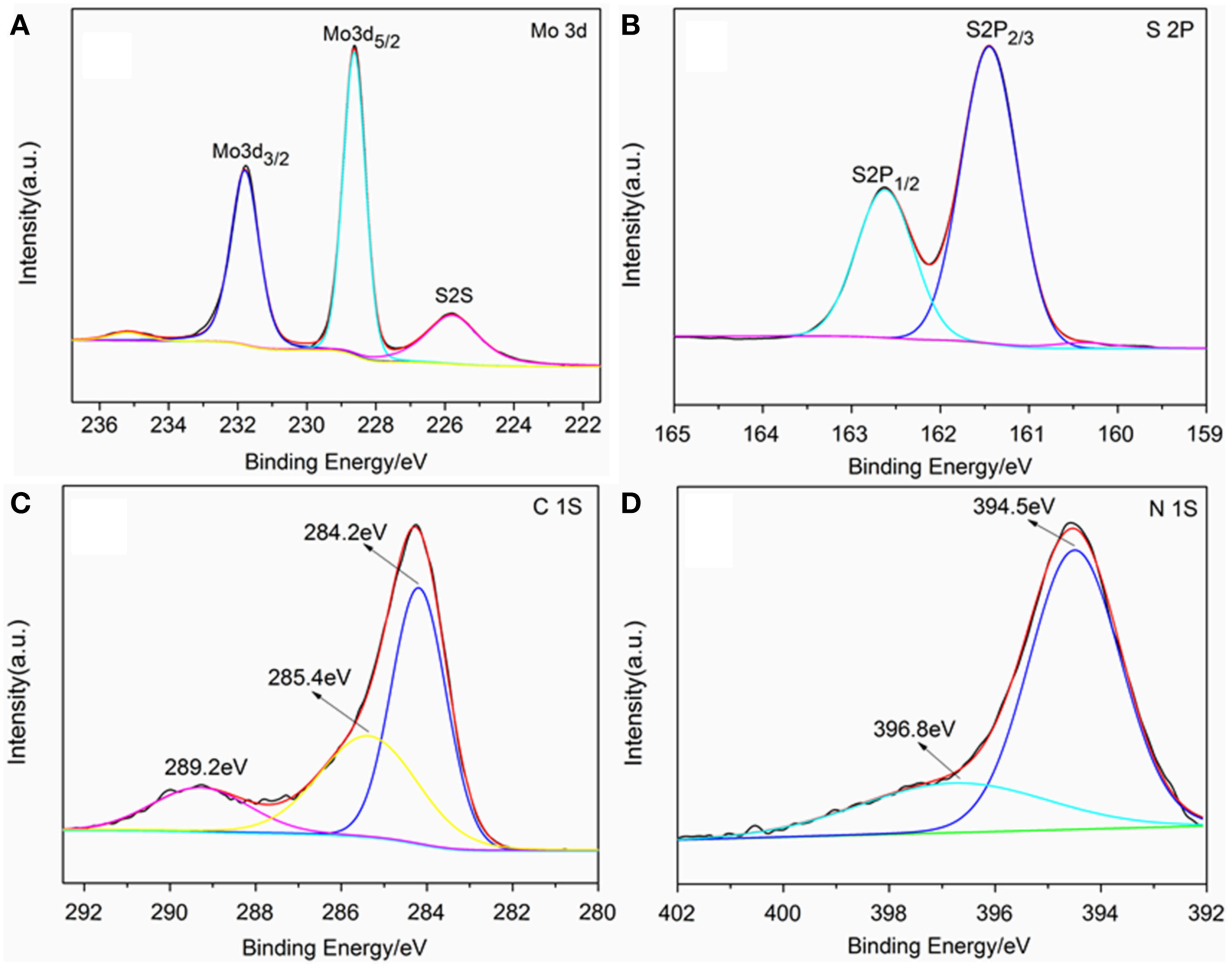
XPS spectrum of TNCT@MoS_2_. Mo 3d spectrum **(A)**, S 2p spectrum **(B)**, C 1s spectrum **(C)**, and N 1S spectrum **(D)**.

The chemical state of surface species of the samples was further determined by X-ray photoelectron spectroscopy (XPS). The survey XPS spectra showed that Mo, S, N, C, and O elements existed in the surface layer of the as-papered nanostructures (Supplementary Figure 2). Two peaks located at 228.62 and 231.79 eV are attributed to Mo 3d5/2 and Mo 3d3/2, respectively (Figure 3B; Wang et al., 2017). The peak located at 161.8 eV corresponds to the S 2p3/2 and another one located at 162.7 eV was assigned to S 2p1/2 (Figure 3C; Hu et al., 2014). Three peaks were observed in the C 1s binding region peaks centered at 284.2, 285.4 and 289.2 eV, which were ascribed to graphite-C, -C^*^-C = O and -C^*^ = O groups, respectively (Figure 3D; Christie et al., 1983; Beamson and Briggs, 1993; Witek et al., 1996). Two peaks were observed in the N1s binding region peaks (Figure 3A). The peak centered at 394.5 eV indicated that the doped nitrogen in TNCT was connected with the carbon frame by -N^*^ = N^*^-C bond (Kudo et al., 1986). It is worth noting that another peak for N1s centered at 396.8 eV was ascribed to Mo(-N^*^ = N-)_2_-C (George and Kwarcinski, 1995), which indicated that the MoS_2_ could closely bond with TNCT by the surface -N=N- group.

**Figure 4:**
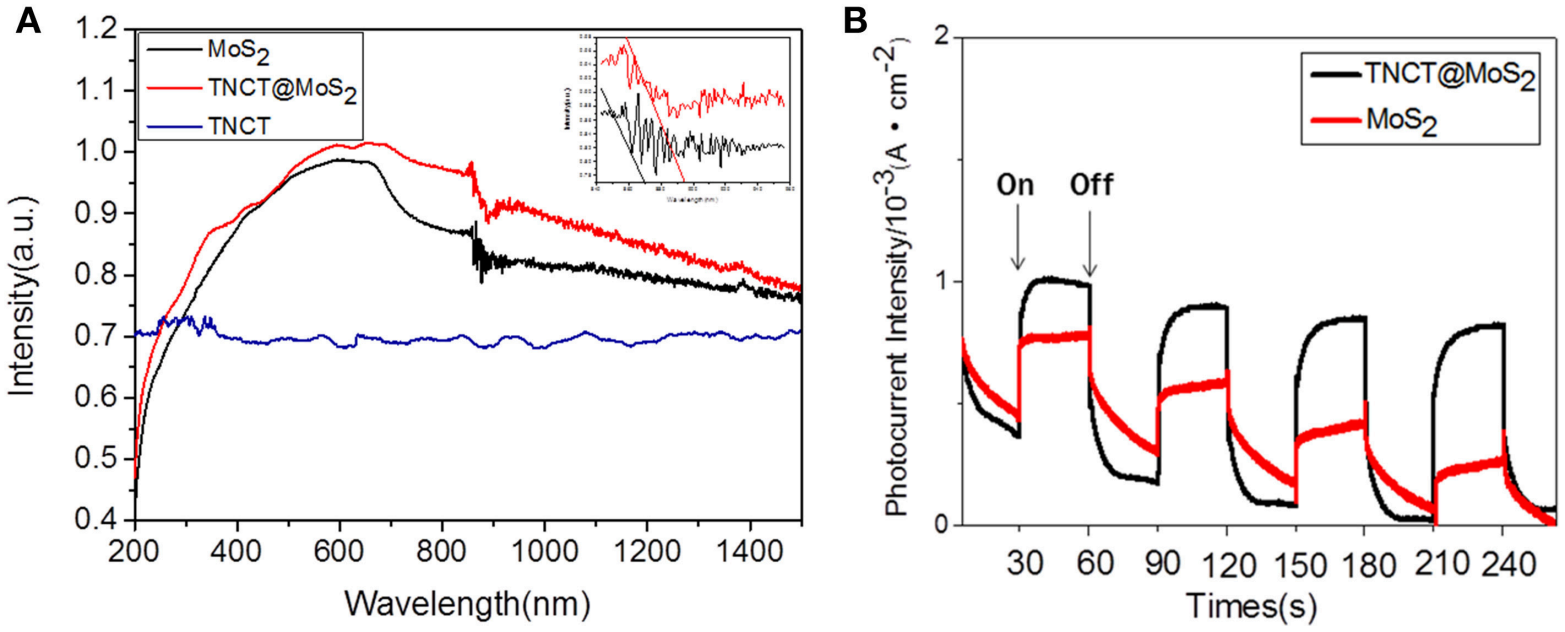
UV-Vis-IR absorbance spectrum **(A)** and photocurrent of the samples **(B)**.

The UV-Vis-IR absorbance spectrum shows that TNCT @MoS_2_ exhibits higher and broader spectral absorption including IR light than that of TNCT and MoS_2_ (Figure 4A). As shown in Figure 4B, TNCT@MoS_2_ obviously shows higher photocurrent density than that of MoS_2_. It indicates that TNCT@ MoS_2_ maybe has better photocatalytic activity.

**Figure 5:**
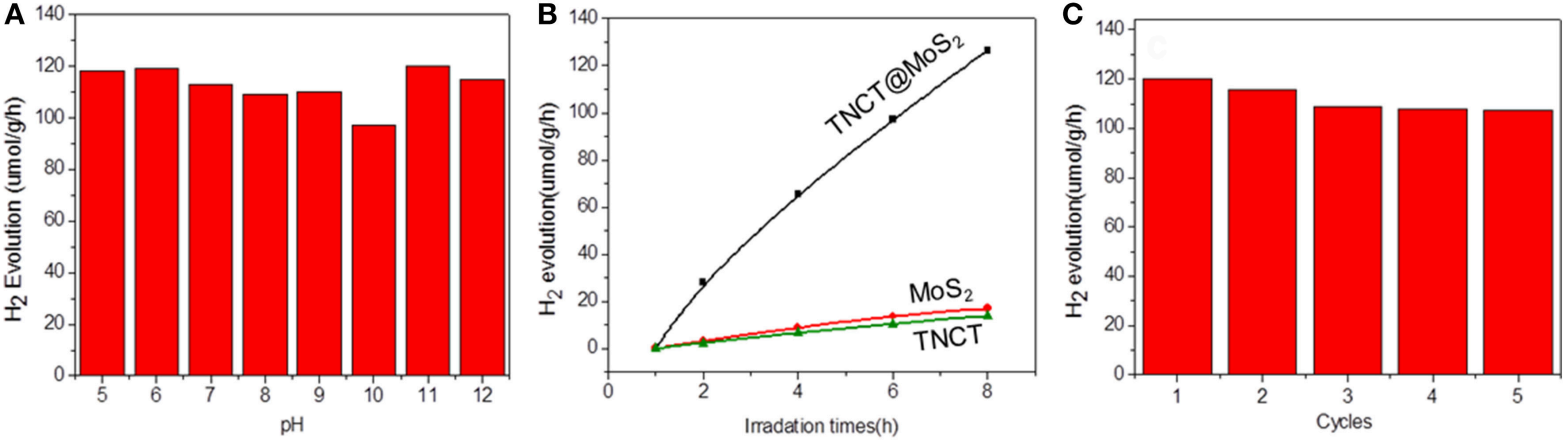
Photocatalytic hydrogen production activity at different pH values **(A)**, hydrogen production data for different samples **(B)**, and recycling performance for TNCT@MoS_2_ **(C)**.

The photocatalytic activity of the as-prepared catalyst was investigated for the photocatalytic water splitting process without addition of any sacrificial agents, which was performed by a 1,000 W xenon lamp source. It was found that the catalyst showed almost the same photocatalytic activity in the wide pH range of 5-11 and pure water (Figure 5A). The optimal hydrogen production rate of TNCT@MoS_2_ was 120 μmol/g·h, which is about 9 times faster than that of pure MoS_2_ (Figure 5B).

For a typical photocatalytic water splitting process, both H_2_ and O_2_ are usually produced from water. However, in this work, whereas this system readily produces hydrogen under illumination, the simultaneous liberation of oxygen is not observed. This phenomenon has been observed in much water splitting systems (Duonghong and Grätzel, 1984; Liu et al., 2012; Zhang et al., 2014). It is because the produced O_2_ or intermediate oxygen species such as OH are readily absorbed by the metal elements such as Mo and W to form steady peroxide complexes, in a result of inhibiting the release of O_2_ (Cui et al., 2015). Herein, although there was no O2 determined in this photocatalytic water splitting system, intermediate OH directly photogenerated from water was detected by the fluorescence probe TA (Yang et al., 2009). The fluorescence spectra of TAOH, which is produced through the oxidation of TA by OH, exhibited a peak at 426 nm, indicating the presence of OH (Supplementary Figure 3). Although the surface of molybdenum sulfide suffer from the produced intermediate active oxygen, the XRD (Figure 2a) and Raman spectroscopy (Supplementary Figure 4) showed that the crystal structure of the material did not change fundamentally and maintain the same photoactivity for five photocatalytic reaction runs (Figure 5C).

**Figure 6:**
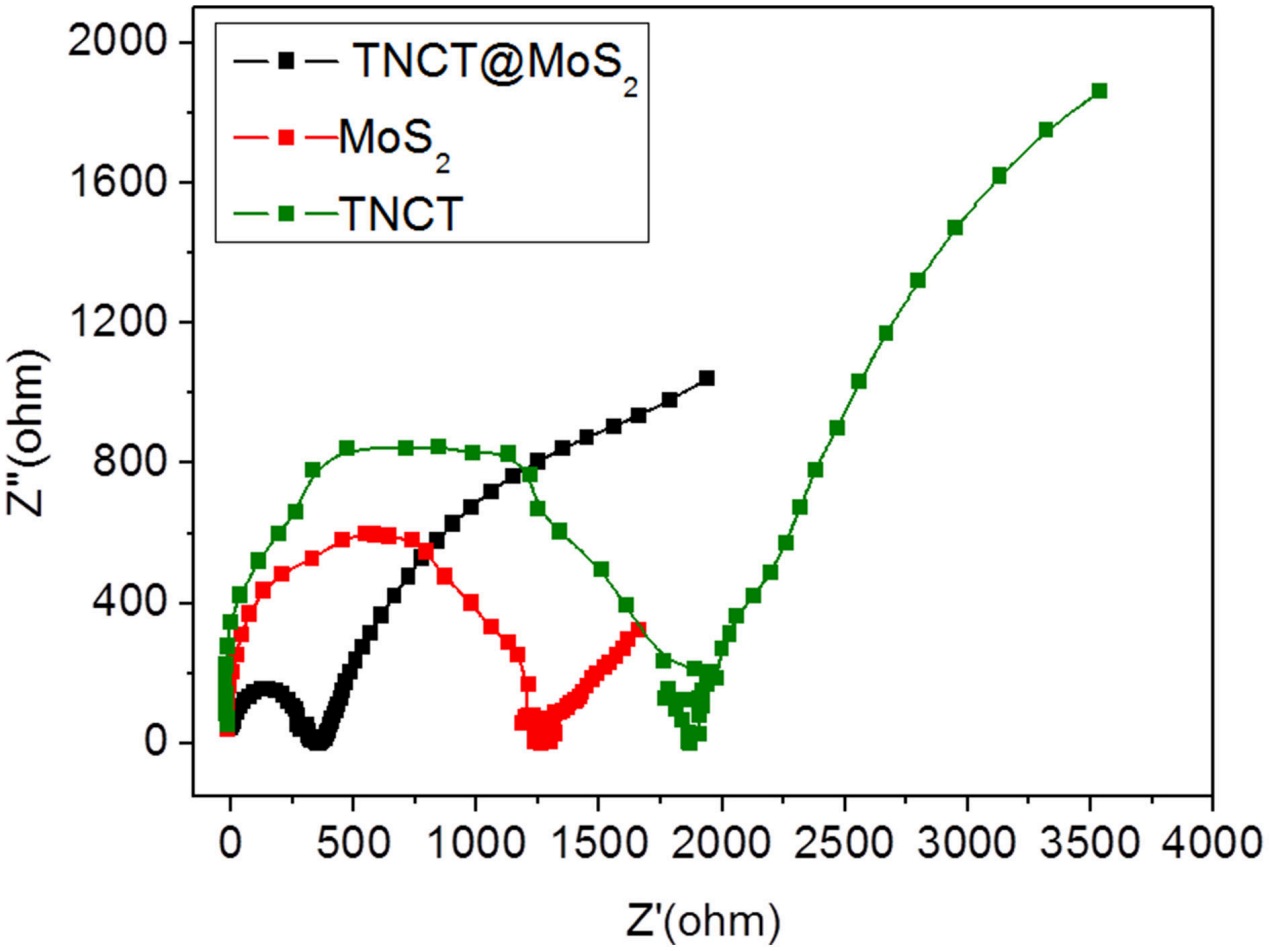
EIS Nynquist plots of the prepared samples.

The excellent photocatalytic performance of TNCT@MoS_2_ is ascribed to the effective separation and transfer of photogenerated carriers. Herein, it was verified by the photoluminescence spectroscopy and photoelectrochemical performance measurements. The photoluminescence (PL) of semiconductors was caused by recombination of photoinduced electrons and holes. If the recombination was suppressed, the PL emission of semiconductors would be quenched.As shown in Supplementary Figure 5, after being combined with TNCT, the PL intensity of MoS_2_ emission peak obviously quenched. It indicated that the photoinduced charge separation efficiency of TNCT@MoS_2_ would improve. The photogenerated carriers' transfer capacity of the as prepared materials was confirmed by electrochemical impedance tests. As shown in Figure 6, TNCT, MoS_2_, and TNCT@MoS_2_ all showed classical semicircular Nyquist diagram. The arc radius of TNCT@MoS_2_ was obviously smaller than that of TNCT and MoS_2_, which indicated that the photogenerated charges in TNCT@MoS_2_ would suffer less resistance. It is ascribed to the fast charge transfer from MoS_2_ nanosheets to carbon tube under full-light irradiation. The formed Mo(-N^*^ = N-)_2_-C bonding connection between MoS_2_ and TNCT maybe acts as carrier transfer bridge (Figure 3D), which will improve the photogenerated carriers separation and transfer efficiency of TNCT@MoS_2_. The detailed mechanism and remained questions would be studied in our future work.

## Conclusions

In summary, a micron-sized three-way nitrogen-doped carbon tube covered with MoS_2_ nanosheets was synthesized and applied in the photocatalytic water splitting without any sacrificial agents for the first time. The micron-sized three-way nitrogen-doped carbon tube is facilely synthesized by the calcination of sponge at 450°C in vacuum. And then the MoS_2_ nanosheets are deposited on the three-way nitrogen-doped carbon tubes by a simple hydrothermal method. Compared with MoS_2_, the TNCT@MoS_2_ heterostructures showed higher H_2_ evolution rate, which may be ascribed to the improved charge separation and transfer efficiency caused by the three-way nitrogen-doped carbon tube. This work may provide a new design idea for the design of low-cost and efficient photocatalysts.

## Author Contributions

GC and BT conceived and designed the experiments. YuZ, YL, PC, HC, and YF performed the experiments. WG, XS, and YiZ analyzed the data. GC and YuZ co-wrote the paper.

### Conflict of Interest Statement

The authors declare that the research was conducted in the absence of any commercial or financial relationships that could be construed as a potential conflict of interest.
