# Supplementary material for: MoS2 Nanosheets Assembled on Three-Way Nitrogen-Doped Carbon Tubes for Photocatalytic Water Splitting
Source: Front Chem. 2019 May 17;7:325. doi: 10.3389/fchem.2019.00325 (PMC6534068; doi:10.3389/fchem.2019.00325)
Supplement: Supplementary file 1 [file Data_Sheet_1.PDF]

Supplementary Material

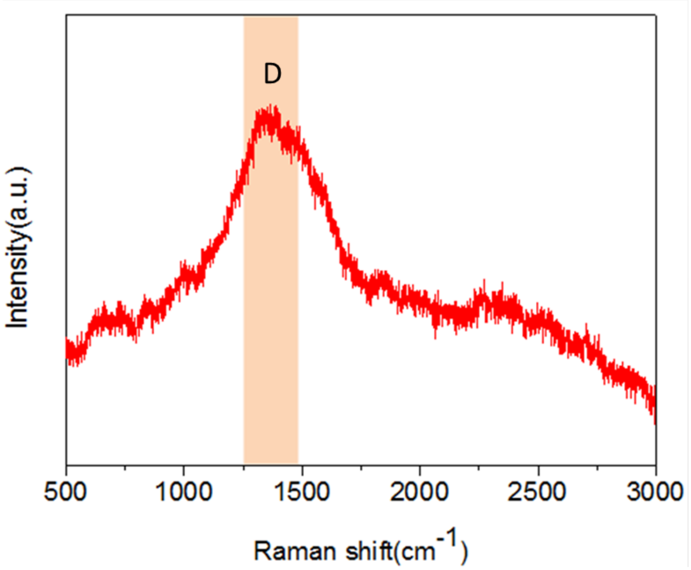

Supplementary Figure 1. Raman spectrum of TNCT

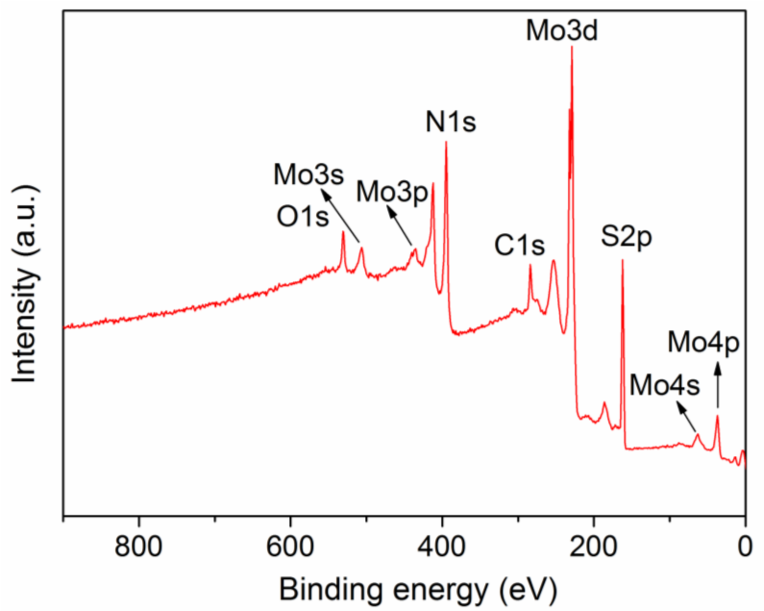

Supplementary Figure 2. Survey XPS spectrum of TNCT@MoS_2

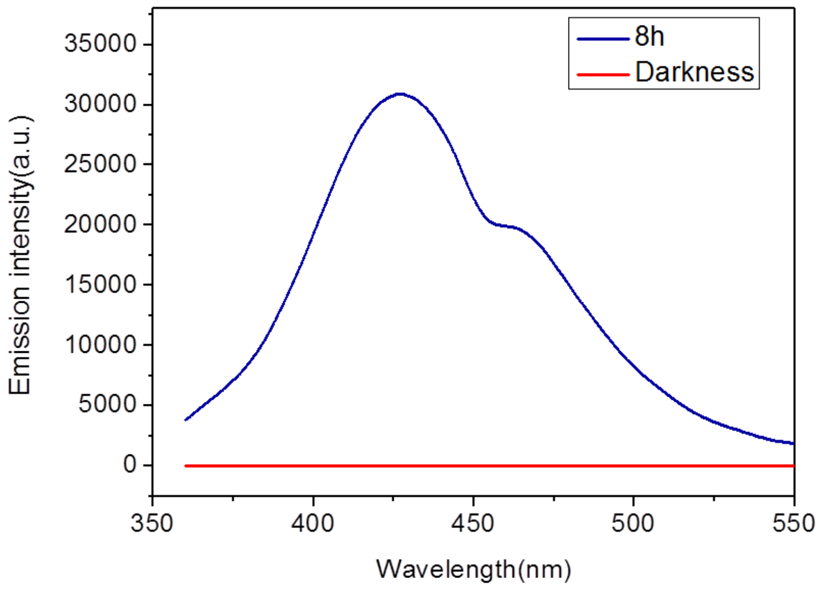

Supplementary Figure 3. Fluorescence spectra of TAOH

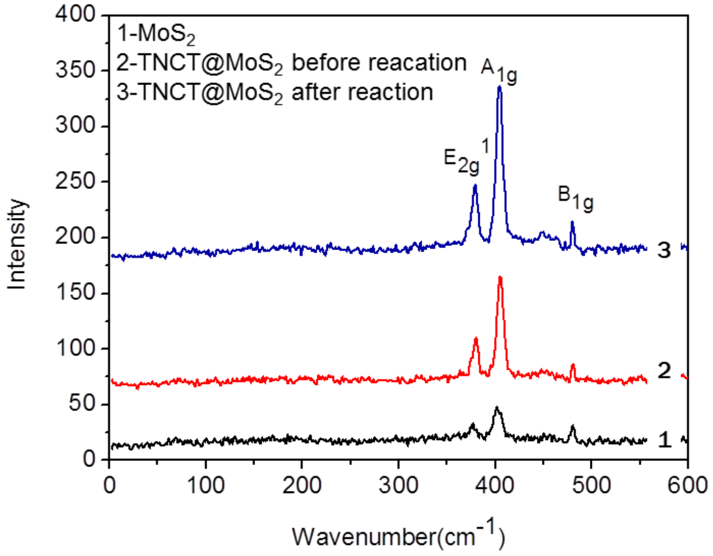

Supplementary Figure 4. Raman spectrum of different samples

The Raman peaks of the catalyst samples centered at 380cm^{-1}, 405.3cm^{-1}, 480.3cm^{-1}, were ascribed to the in-plane vibration of the S-Mo-S atom(E_{2g}^{1}), the out-of-plane stretching vibration of the S atom(A_{1g}), and the characteristic peak position of the MoS_2 (B_{1g}), respectively. It is consistent with the results reported in the literature.

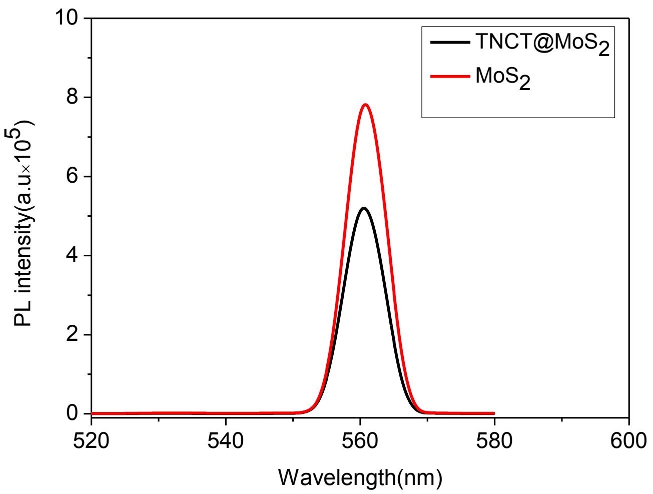

Supplementary Figure 5. Photoluminescence spectrum of different samples
